# Dimethyl­ammonium perchlorate 18-crown-6 monohydrate clathrate

**DOI:** 10.1107/S1600536810023421

**Published:** 2010-06-23

**Authors:** Jia-Zhen Ge, Min-Min Zhao, Ping-Ping Shi

**Affiliations:** a Ordered Matter Science Research Center, College of Chemistry and Chemical, Engineering, Southeast University, Nanjing 211189, People’s Republic of China

## Abstract

The reaction of dimethyl­amine, 18-crown-6, and perchloric acid in methanol yields the title compound, C_2_H_8_N^+^·ClO_4_^−^·C_12_H_24_O_6_·H_2_O. The dimethyl­ammonium cation and the water mol­ecule inter­act with the 18-crown-6 unit: N—H⋯O hydrogen bonds are formed between the ammonium NH_2_^+^ group and four O atoms of the crown ether, while the water mol­ecule on the other side of 18-crown-6 ring forms O—H⋯O hydrogen bonds with two other O atoms of the crown ether. All conventional donors and acceptors in the cations are thus engaged in hydrogen bonding. The ClO_4_^−^ anion is disordered over two sites, and occupancies for the disordered O atoms were fixed at 0.5. In the crystal, the cations and anions are arranged in alternating layers.

## Related literature

For the similar structure, 18-crown-6 clathrate, see: Pedersen (1967 ▶). For the ferroelectric properties of related materials, see: Fu *et al.* (2007 ▶); Ye *et al.* (2009 ▶); Zhang *et al.* (2009 ▶). 
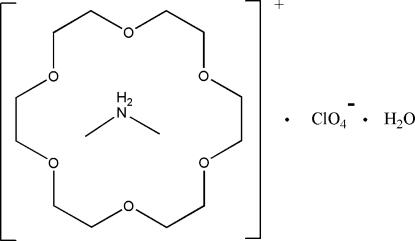


## Experimental

### 

#### Crystal data

C_2_H_8_N^+^·ClO_4_^−^·C_12_H_24_O_6_·H_2_O
*M* *_r_* = 427.87
Orthorhombic, 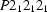

*a* = 10.684 (2) Å
*b* = 13.954 (3) Å
*c* = 14.583 (3) Å
*V* = 2174.1 (8) Å^3^
*Z* = 4
Mo *K*α radiation
μ = 0.23 mm^−1^
*T* = 293 K
0.4 × 0.3 × 0.2 mm

#### Data collection

Rigaku SCXmini diffractometer
Absorption correction: multi-scan *CrystalClear* (Rigaku, 2005 ▶) *T*_min_ = 0.955, *T*_max_ = 0.955
22574 measured reflections
4973 independent reflections
2814 reflections with *I* > 2σ(*I*)
*R*_int_ = 0.070

#### Refinement

*R*[*F* ^2^ > 2σ(*F* ^2^)] = 0.058
*wR*(*F* ^2^) = 0.171
*S* = 1.00
4973 reflections
283 parameters
51 restraints
H-atom parameters constrained
Δρ_max_ = 0.29 e Å^−3^
Δρ_min_ = −0.15 e Å^−3^
Absolute structure: Flack (1983 ▶), 2164 Friedel pairs
Flack parameter: 0.02 (10)

### 

Data collection: *CrystalClear* (Rigaku, 2005 ▶); cell refinement: *CrystalClear*; data reduction: *CrystalClear*; program(s) used to solve structure: *SHELXS97* (Sheldrick, 2008 ▶); program(s) used to refine structure: *SHELXL97* (Sheldrick, 2008 ▶); molecular graphics: *SHELXTL* (Sheldrick, 2008 ▶); software used to prepare material for publication: *PRPKAPPA* (Ferguson, 1999 ▶).

## Supplementary Material

Crystal structure: contains datablocks I, New_Global_Publ_Block. DOI: 10.1107/S1600536810023421/bh2290sup1.cif

Structure factors: contains datablocks I. DOI: 10.1107/S1600536810023421/bh2290Isup2.hkl

Additional supplementary materials:  crystallographic information; 3D view; checkCIF report

## Figures and Tables

**Table 1:** Hydrogen-bond geometry (Å, °)

*D*—H⋯*A*	*D*—H	H⋯*A*	*D*⋯*A*	*D*—H⋯*A*
N1—H1*AA*⋯O6	1.05	2.00	2.979 (4)	154
N1—H1*BB*⋯O3	0.86	2.14	2.979 (4)	164
O20—H20*B*⋯O4^i^	0.90	2.12	3.013 (4)	171
O20—H20*A*⋯O1^i^	0.76	2.21	2.927 (3)	158

Symmetry code: (i) 
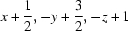
.

**Fig. 1.**
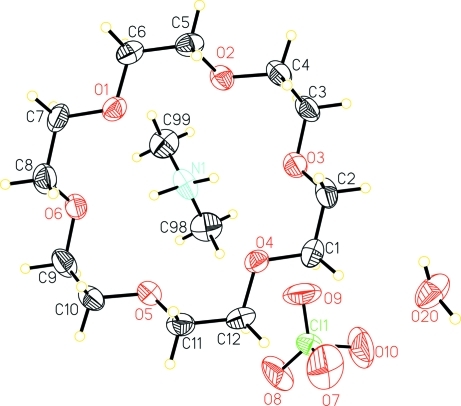
The molecular structure of the title compound, with the atomic numbering scheme. Displacement ellipsoids are drawn at the 30% probability level and a single orientation for the disordered perchlorate anion is shown.

**Fig. 2.**
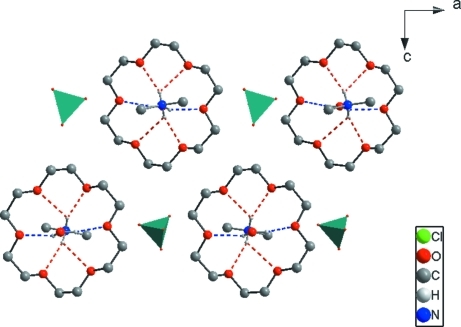
A view of the packing of the title compound, stacking along the b axis. Dashed lines indicate hydrogen bonds.

## supplementary crystallographic information

### Comment

The crown ethers are of a great interest since their discovery by Pedersen (1967). The ability of these macrocycles to form non-covalent, H-bonding complexes with ammonium cations has been actively investigated. Both the size of the crown ether and the nature of the ammonium cation (NH_4_^+^, *R*NH_3_^+^, *etc*) can influence on the stoichiometry and stability of these host-guest complexes. The host molecules combine with the guest species by intermolecular interactions, and if the host molecule has some specific sites, it is easy to realize high selectivity in ion or molecular recognition. 18-crown-6 has the highest affinity for ammonium cations *R*NH_3_^+^, and most studies of 18-crown-6 and its derivatives invariably showed the 1:1 stoichiometry with *R*NH_3_^+^ cations.

The title compound dielectric permittivity was tested to systematically investigate the ferroelectric phase transitions of these materials (Fu *et al.*, 2007; Ye *et al.*, 2009; Zhang *et al.*, 2009). The title compound has no dielectric anomalies, with the relative permittivity at 1 MHz being in the range 4–5 between 80 and 330 K (m.p. is 353 K), suggesting that no phase transition occurred within the measured temperature range.

The title compound is composed of cation [(CH_3_)_2_NH_2_(18-Crown-6)]^+^, one isolated anion (ClO_4_)^-^, and one lattice water molecule (Fig 1). The protonated dimethylamine [(CH_3_)_2_NH_2_]^+^ and 18-crown-6 form a supramolecular structure through N—H···O hydrogen bonds between the ammonium NH_2_^+^ group and four O atoms of the crown ether. The water molecule on the other side of the 18-crown-6 ring forms O—H···O hydrogen bonds between the O—H groups of water and two other O atoms of the crown ether. The intramolecular N—H···O hydrogen bond lengths are within the usual range: 2.979 (4)–3.285 (4) Å, and the intramolecular O—H···O hydrogen bond lengths are from 2.927 (3) to 3.013 (4) Å. The crown ring shows severe distortions. The six O atoms of the crown ether take the approximate "boat-shape" conformation. The four O atoms bonding to the NH_2_^+^ cation lie on the bottom of the boat, while the other two O atoms bonded to the water molecule lie on the head and tail of the boat.

The (ClO_4_)^-^ anion is disordered, as detectable from the large displacement parameters for O atoms and short Cl—O bond lengths. The disorder was modelled with two sites with equal occupancies for O atoms, and the geometry of the anion was regularized through soft restraints.

Fig. 2 shows a view of the crystal structure down the *b* axis. The cations [(CH_3_)_2_NH_2_(18-Crown-6)]^+^ are arranged into layers almost parallel to (101). The anions (ClO_4_)^-^ are placed in the voids formed by the cations. The title compound is stabilized by intramolecular N—H···O and O—H···O hydrogen bonds, as above described, but no intermolecular hydrogen bonds are observed.

### Experimental

Dimethylamine (2 mmol, 0.09 g) and an excess of perchloric acid (3 mmol, 0.302 g) were dissolved in methanol. Then, 18-crown-6 (2 mmol, 0.528 g) was added to the mixture. The precipitate was filtered and washed with a small amount of methanol. Single crystals suitable for X-ray diffraction were obtained by slow evaporation of a methanol solution at room temperature over two days.

### Refinement

All C-bonded H atoms were placed geometrically, with the C—H distances ranging from 0.96 to 0.97 Å. Isotropic displacement parameters were calculated as *U*_iso_(H) = 1.2*U*_eq_(C) for methylene groups and *U*_iso_(H) = 1.5*U*_eq_(C) for methyl groups. H atoms bonded to N and O atoms were found in a difference map and refined as riding atoms and with *U*_iso_(H) = 1.5*U*_eq_(carrier atom). O atoms for the perchlorate ion were splited over two sites, with equal occupancies fixed to 0.5. Cl—O bond lengths were restrained, as well as displacement parameters for disordered O atoms (51 restraints).

### Crystal data

**Table:**

C_2_H_8_N^+^·ClO_4_^−^·C_12_H_24_O_6_·H_2_O	*D*_x_ = 1.307 Mg m^−^^3^
*M**_r_* = 427.87	Melting point: 353 K
Orthorhombic, *P*2_1_2_1_2_1_	Mo *K*α radiation, λ = 0.71073 Å
Hall symbol: P 2ac 2ab	Cell parameters from 2814 reflections
*a* = 10.684 (2) Å	θ = 3.2–27.5°
*b* = 13.954 (3) Å	µ = 0.23 mm^−^^1^
*c* = 14.583 (3) Å	*T* = 293 K
*V* = 2174.1 (8) Å^3^	Block, white
*Z* = 4	0.4 × 0.3 × 0.2 mm
*F*(000) = 920	

### Data collection

**Table:**

Rigaku SCXmini diffractometer	4973 independent reflections
Radiation source: fine-focus sealed tube	2814 reflections with *I* > 2σ(*I*)
graphite	*R*_int_ = 0.070
Detector resolution: 13.6612 pixels mm^-1^	θ_max_ = 27.5°, θ_min_ = 3.2°
ω scans	*h* = −13→13
Absorption correction: multi-scan *CrystalClear* (Rigaku, 2005)	*k* = −18→18
*T*_min_ = 0.955, *T*_max_ = 0.955	*l* = −18→18
22574 measured reflections	

### Refinement

**Table:**

Refinement on *F*^2^	Secondary atom site location: difference Fourier map
Least-squares matrix: full	Hydrogen site location: inferred from neighbouring sites
*R*[*F*^2^ > 2σ(*F*^2^)] = 0.058	H-atom parameters constrained
*wR*(*F*^2^) = 0.171	*w* = 1/[σ^2^(*F*_o_^2^) + (0.0794*P*)^2^ + 0.2447*P*] where *P* = (*F*_o_^2^ + 2*F*_c_^2^)/3
*S* = 1.00	(Δ/σ)_max_ = 0.007
4973 reflections	Δρ_max_ = 0.29 e Å^−^^3^
283 parameters	Δρ_min_ = −0.15 e Å^−^^3^
51 restraints	Absolute structure: Flack (1983), 2168 Friedel pairs
0 constraints	Flack parameter: 0.02 (10)
Primary atom site location: structure-invariant direct methods	

### Fractional atomic coordinates and isotropic or equivalent isotropic displacement parameters (Å^2^)

**Table:**

	*x*	*y*	*z*	*U*_iso_*/*U*_eq_	Occ. (<1)
Cl1	0.98649 (9)	0.31614 (6)	0.61639 (6)	0.0683 (3)	
O1	0.26118 (19)	0.62910 (16)	0.62120 (17)	0.0615 (6)	
O4	0.72647 (19)	0.61810 (16)	0.58783 (16)	0.0580 (6)	
O5	0.6370 (2)	0.58013 (18)	0.76505 (16)	0.0661 (7)	
O2	0.3466 (2)	0.56628 (17)	0.44918 (16)	0.0631 (6)	
O3	0.6070 (2)	0.51865 (17)	0.43975 (16)	0.0659 (7)	
O6	0.3742 (2)	0.54342 (19)	0.77918 (17)	0.0686 (7)	
C6	0.1812 (3)	0.6189 (3)	0.5450 (3)	0.0666 (10)	
H6A	0.1124	0.6640	0.5498	0.080*	
H6B	0.1466	0.5547	0.5439	0.080*	
C12	0.8060 (3)	0.6151 (3)	0.6658 (3)	0.0690 (10)	
H12A	0.8769	0.6575	0.6567	0.083*	
H12B	0.8375	0.5506	0.6745	0.083*	
C5	0.2518 (3)	0.6370 (3)	0.4595 (3)	0.0655 (9)	
H5A	0.1954	0.6348	0.4074	0.079*	
H5B	0.2895	0.7001	0.4618	0.079*	
C7	0.1976 (3)	0.6136 (3)	0.7052 (3)	0.0729 (11)	
H7A	0.1592	0.5507	0.7050	0.088*	
H7B	0.1320	0.6611	0.7126	0.088*	
C11	0.7341 (4)	0.6456 (3)	0.7479 (3)	0.0683 (10)	
H11A	0.7893	0.6487	0.8006	0.082*	
H11B	0.6993	0.7090	0.7378	0.082*	
C3	0.5202 (4)	0.5127 (3)	0.3647 (2)	0.0742 (11)	
H3A	0.4825	0.4494	0.3642	0.089*	
H3B	0.5655	0.5207	0.3075	0.089*	
C2	0.7006 (4)	0.5896 (3)	0.4281 (3)	0.0743 (11)	
H2A	0.6621	0.6525	0.4247	0.089*	
H2B	0.7456	0.5784	0.3714	0.089*	
C4	0.4200 (4)	0.5857 (3)	0.3699 (2)	0.0745 (10)	
H4A	0.4563	0.6493	0.3741	0.089*	
H4B	0.3681	0.5830	0.3154	0.089*	
C10	0.5660 (4)	0.6078 (3)	0.8436 (3)	0.0758 (11)	
H10A	0.5341	0.6724	0.8357	0.091*	
H10B	0.6185	0.6066	0.8979	0.091*	
C9	0.4614 (4)	0.5398 (3)	0.8540 (3)	0.0822 (12)	
H9A	0.4948	0.4754	0.8587	0.099*	
H9B	0.4174	0.5539	0.9106	0.099*	
C1	0.7897 (3)	0.5862 (3)	0.5074 (3)	0.0697 (10)	
H1A	0.8195	0.5211	0.5161	0.084*	
H1B	0.8612	0.6270	0.4952	0.084*	
C8	0.2889 (4)	0.6209 (3)	0.7833 (3)	0.0818 (12)	
H8A	0.3340	0.6810	0.7795	0.098*	
H8B	0.2441	0.6194	0.8412	0.098*	
O7	1.0420 (16)	0.4009 (9)	0.6144 (13)	0.197 (9)	0.50
O8	1.0022 (13)	0.2735 (7)	0.6991 (6)	0.140 (5)	0.50
O9	0.8759 (8)	0.3033 (10)	0.5711 (8)	0.152 (4)	0.50
O10	1.0765 (12)	0.2850 (9)	0.5452 (6)	0.172 (4)	0.50
O7'	0.9855 (17)	0.4068 (8)	0.6012 (13)	0.186 (8)	0.50
O8'	0.960 (2)	0.2425 (11)	0.5730 (13)	0.276 (10)	0.50
O9'	1.072 (2)	0.2849 (18)	0.6660 (13)	0.313 (13)	0.50
O10'	0.8799 (10)	0.3385 (8)	0.6824 (10)	0.184 (5)	0.50
N1	0.4833 (3)	0.45369 (18)	0.61194 (19)	0.0711 (9)	
H1AA	0.4615	0.5025	0.6643	0.107*	
H1BB	0.5139	0.4836	0.5651	0.107*	
O20	0.9938 (2)	0.7613 (2)	0.3947 (2)	0.0993 (10)	
H20A	0.9312	0.7809	0.3782	0.149*	
H20B	1.0611	0.8000	0.3942	0.149*	
C98	0.5941 (4)	0.3948 (3)	0.6317 (4)	0.0945 (14)	
H98A	0.6657	0.4356	0.6395	0.142*	
H98B	0.6087	0.3515	0.5817	0.142*	
H98C	0.5803	0.3589	0.6870	0.142*	
C99	0.3696 (4)	0.3968 (3)	0.6010 (3)	0.0905 (13)	
H99A	0.3009	0.4383	0.5861	0.136*	
H99B	0.3519	0.3636	0.6572	0.136*	
H99C	0.3813	0.3511	0.5526	0.136*	

### Atomic displacement parameters (Å^2^)

**Table:**

	*U*^11^	*U*^22^	*U*^33^	*U*^12^	*U*^13^	*U*^23^
Cl1	0.0842 (6)	0.0557 (5)	0.0649 (5)	−0.0032 (5)	−0.0152 (5)	0.0061 (5)
O1	0.0462 (11)	0.0691 (15)	0.0693 (14)	−0.0068 (10)	0.0032 (12)	−0.0023 (13)
O4	0.0470 (12)	0.0637 (14)	0.0632 (14)	0.0053 (11)	−0.0018 (11)	0.0033 (11)
O5	0.0686 (15)	0.0762 (17)	0.0535 (14)	−0.0004 (14)	−0.0026 (13)	−0.0064 (12)
O2	0.0706 (15)	0.0653 (14)	0.0533 (13)	0.0008 (12)	0.0002 (12)	0.0075 (12)
O3	0.0718 (15)	0.0691 (15)	0.0569 (15)	0.0070 (13)	0.0017 (13)	0.0003 (13)
O6	0.0656 (15)	0.0867 (19)	0.0535 (14)	−0.0118 (14)	0.0089 (13)	−0.0105 (13)
C6	0.0490 (19)	0.068 (2)	0.083 (3)	0.0046 (17)	−0.005 (2)	0.007 (2)
C12	0.0472 (18)	0.073 (2)	0.087 (3)	−0.0077 (17)	−0.015 (2)	0.000 (2)
C5	0.059 (2)	0.061 (2)	0.076 (2)	0.0041 (18)	−0.011 (2)	0.0118 (19)
C7	0.0488 (19)	0.089 (3)	0.081 (3)	−0.0001 (19)	0.019 (2)	−0.014 (2)
C11	0.061 (2)	0.072 (2)	0.071 (2)	−0.0045 (19)	−0.013 (2)	−0.0100 (19)
C3	0.092 (3)	0.087 (3)	0.0432 (19)	0.016 (2)	−0.003 (2)	−0.0128 (18)
C2	0.089 (3)	0.079 (3)	0.054 (2)	0.012 (2)	0.018 (2)	0.008 (2)
C4	0.087 (2)	0.090 (3)	0.046 (2)	−0.002 (2)	−0.005 (2)	0.001 (2)
C10	0.083 (2)	0.095 (3)	0.049 (2)	−0.008 (2)	−0.008 (2)	−0.008 (2)
C9	0.105 (3)	0.095 (3)	0.047 (2)	−0.015 (3)	0.004 (2)	0.0011 (19)
C1	0.058 (2)	0.073 (3)	0.078 (3)	0.0017 (19)	0.017 (2)	0.006 (2)
C8	0.081 (3)	0.093 (3)	0.071 (3)	−0.011 (3)	0.016 (2)	−0.026 (2)
O7	0.232 (15)	0.149 (12)	0.209 (14)	−0.154 (12)	0.056 (12)	−0.047 (10)
O8	0.219 (14)	0.133 (7)	0.067 (4)	−0.045 (10)	−0.007 (6)	0.056 (4)
O9	0.089 (5)	0.204 (11)	0.161 (9)	−0.010 (6)	−0.055 (6)	−0.045 (9)
O10	0.192 (9)	0.221 (12)	0.103 (6)	0.045 (9)	0.057 (6)	−0.033 (7)
O7'	0.269 (19)	0.070 (7)	0.220 (14)	0.042 (9)	0.012 (14)	0.085 (8)
O8'	0.36 (2)	0.156 (11)	0.31 (2)	−0.123 (13)	0.08 (2)	−0.159 (14)
O9'	0.295 (19)	0.40 (3)	0.248 (19)	0.26 (2)	−0.030 (16)	0.118 (17)
O10'	0.127 (7)	0.174 (9)	0.251 (13)	0.008 (7)	0.059 (8)	−0.045 (9)
N1	0.121 (3)	0.0387 (14)	0.0533 (16)	0.0026 (17)	0.009 (2)	0.0043 (12)
O20	0.0592 (15)	0.0859 (19)	0.153 (3)	0.0080 (15)	0.0083 (19)	0.0404 (18)
C98	0.103 (3)	0.065 (2)	0.115 (4)	−0.013 (2)	−0.009 (3)	−0.008 (3)
C99	0.092 (3)	0.070 (2)	0.110 (4)	0.007 (2)	0.002 (3)	0.013 (3)

### Geometric parameters (Å, °)

**Table:**

Cl1—O8'	1.239 (9)	C11—H11A	0.9700
Cl1—O9'	1.246 (13)	C11—H11B	0.9700
Cl1—O7'	1.285 (9)	C3—C4	1.481 (5)
Cl1—O7	1.324 (9)	C3—H3A	0.9700
Cl1—O8	1.356 (8)	C3—H3B	0.9700
Cl1—O9	1.365 (7)	C2—C1	1.498 (5)
Cl1—O10	1.480 (8)	C2—H2A	0.9700
Cl1—O10'	1.523 (10)	C2—H2B	0.9700
O1—C6	1.409 (4)	C4—H4A	0.9700
O1—C7	1.418 (4)	C4—H4B	0.9700
O4—C12	1.420 (4)	C10—C9	1.474 (6)
O4—C1	1.425 (4)	C10—H10A	0.9700
O5—C11	1.405 (4)	C10—H10B	0.9700
O5—C10	1.427 (4)	C9—H9A	0.9700
O2—C5	1.422 (4)	C9—H9B	0.9700
O2—C4	1.422 (4)	C1—H1A	0.9700
O3—C2	1.418 (5)	C1—H1B	0.9700
O3—C3	1.437 (4)	C8—H8A	0.9700
O6—C8	1.415 (5)	C8—H8B	0.9700
O6—C9	1.435 (4)	N1—C99	1.460 (5)
C6—C5	1.479 (5)	N1—C98	1.469 (5)
C6—H6A	0.9700	N1—H1AA	1.0493
C6—H6B	0.9700	N1—H1BB	0.8642
C12—C11	1.485 (5)	O20—H20A	0.7616
C12—H12A	0.9700	O20—H20B	0.9001
C12—H12B	0.9700	C98—H98A	0.9600
C5—H5A	0.9700	C98—H98B	0.9600
C5—H5B	0.9700	C98—H98C	0.9600
C7—C8	1.502 (5)	C99—H99A	0.9600
C7—H7A	0.9700	C99—H99B	0.9600
C7—H7B	0.9700	C99—H99C	0.9600
			
O7—Cl1—O8	110.8 (9)	O3—C2—H2A	109.8
O7—Cl1—O9	119.6 (10)	C1—C2—H2A	109.8
O8—Cl1—O9	118.7 (8)	O3—C2—H2B	109.8
O7—Cl1—O10	87.5 (10)	C1—C2—H2B	109.8
O8—Cl1—O10	114.5 (8)	H2A—C2—H2B	108.2
O9—Cl1—O10	100.7 (7)	O2—C4—C3	108.0 (3)
O8'—Cl1—O9'	99.9 (16)	O2—C4—H4A	110.1
O8'—Cl1—O7'	136.6 (14)	C3—C4—H4A	110.1
O9'—Cl1—O7'	116.8 (15)	O2—C4—H4B	110.1
O8'—Cl1—O10'	109.0 (10)	C3—C4—H4B	110.1
O9'—Cl1—O10'	104.7 (9)	H4A—C4—H4B	108.4
O7'—Cl1—O10'	84.3 (10)	O5—C10—C9	108.2 (3)
C6—O1—C7	112.1 (2)	O5—C10—H10A	110.1
C12—O4—C1	111.5 (2)	C9—C10—H10A	110.1
C11—O5—C10	111.0 (3)	O5—C10—H10B	110.1
C5—O2—C4	110.3 (3)	C9—C10—H10B	110.1
C2—O3—C3	113.9 (3)	H10A—C10—H10B	108.4
C8—O6—C9	114.4 (3)	O6—C9—C10	113.1 (3)
O1—C6—C5	109.8 (3)	O6—C9—H9A	109.0
O1—C6—H6A	109.7	C10—C9—H9A	109.0
C5—C6—H6A	109.7	O6—C9—H9B	109.0
O1—C6—H6B	109.7	C10—C9—H9B	109.0
C5—C6—H6B	109.7	H9A—C9—H9B	107.8
H6A—C6—H6B	108.2	O4—C1—C2	108.9 (3)
O4—C12—C11	109.1 (3)	O4—C1—H1A	109.9
O4—C12—H12A	109.9	C2—C1—H1A	109.9
C11—C12—H12A	109.9	O4—C1—H1B	109.9
O4—C12—H12B	109.9	C2—C1—H1B	109.9
C11—C12—H12B	109.9	H1A—C1—H1B	108.3
H12A—C12—H12B	108.3	O6—C8—C7	109.5 (3)
O2—C5—C6	109.6 (3)	O6—C8—H8A	109.8
O2—C5—H5A	109.8	C7—C8—H8A	109.8
C6—C5—H5A	109.8	O6—C8—H8B	109.8
O2—C5—H5B	109.8	C7—C8—H8B	109.8
C6—C5—H5B	109.8	H8A—C8—H8B	108.2
H5A—C5—H5B	108.2	C99—N1—C98	112.8 (3)
O1—C7—C8	109.5 (3)	C99—N1—H1AA	104.3
O1—C7—H7A	109.8	C98—N1—H1AA	113.5
C8—C7—H7A	109.8	C99—N1—H1BB	119.3
O1—C7—H7B	109.8	C98—N1—H1BB	97.0
C8—C7—H7B	109.8	H1AA—N1—H1BB	110.2
H7A—C7—H7B	108.2	H20A—O20—H20B	118.8
O5—C11—C12	109.8 (3)	N1—C98—H98A	109.5
O5—C11—H11A	109.7	N1—C98—H98B	109.5
C12—C11—H11A	109.7	H98A—C98—H98B	109.5
O5—C11—H11B	109.7	N1—C98—H98C	109.5
C12—C11—H11B	109.7	H98A—C98—H98C	109.5
H11A—C11—H11B	108.2	H98B—C98—H98C	109.5
O3—C3—C4	112.8 (3)	N1—C99—H99A	109.5
O3—C3—H3A	109.0	N1—C99—H99B	109.5
C4—C3—H3A	109.0	H99A—C99—H99B	109.5
O3—C3—H3B	109.0	N1—C99—H99C	109.5
C4—C3—H3B	109.0	H99A—C99—H99C	109.5
H3A—C3—H3B	107.8	H99B—C99—H99C	109.5
O3—C2—C1	109.5 (3)		

### Hydrogen-bond geometry (Å, °)

**Table:**

*D*—H···*A*	*D*—H	H···*A*	*D*···*A*	*D*—H···*A*
N1—H1AA···O6	1.05	2.00	2.979 (4)	154
N1—H1AA···O5	1.05	2.62	3.285 (4)	121
N1—H1BB···O3	0.86	2.14	2.979 (4)	164
O20—H20B···O4^i^	0.90	2.12	3.013 (4)	171
O20—H20A···O1^i^	0.76	2.21	2.927 (3)	158

Symmetry codes: (i) *x*+1/2, −*y*+3/2, −*z*+1.
